# Culture without copying or selection

**DOI:** 10.1017/ehs.2021.47

**Published:** 2021-11-04

**Authors:** Alberto Acerbi, Mathieu Charbonneau, Helena Miton, Thom Scott-Phillips

**Affiliations:** 1 Centre for Culture and Evolution, Division of Psychology, Brunel University, London, UB8 3PH, UK; 2 Faculté de Gouvernance, Sciences Économiques et Sociales, Université Mohammed VI Polytechnique, Rabat-Salé, Morocco; 3 Santa Fe Institute, 1399 Hyde Park Rd, Santa Fe, NM 87501, US; 4 Department of Cognitive Science, Central European University, Október 6. u. 7, 1051, Hungary; 5 Department of Anthropology, South Rd, Durham DH1 3LE, UK

**Keywords:** culture, cultural evolution, cultural transmission, convergent transformation, cultural attraction, individual based model

## Abstract

Typical examples of cultural phenomena all exhibit a degree of similarity across time and space at the level of the population. As such, a fundamental question for any science of culture is, what ensures this stability in the first place? Here we focus on the evolutionary and stabilising role of ‘convergent transformation’, in which one item causes the production of another item whose form tends to deviate from the original in a directed, non-random way. We present a series of stochastic models of cultural evolution investigating its effects. The results show that cultural stability can emerge and be maintained by virtue of convergent transformation alone, in the absence of any form of copying or selection process. We show how high-fidelity copying and convergent transformation need not be opposing forces, and can jointly contribute to cultural stability. We finally analyse how non-random transformation and high-fidelity copying can have different evolutionary signatures at population level, and hence how their distinct effects can be distinguished in empirical records. Collectively, these results supplement existing approaches to cultural evolution based on the Darwinian analogy, while also providing formal support for other frameworks – such as Cultural Attraction Theory – that entail its further loosening.

**Social media summary:**

Culture can be produced and maintained by convergent transformation, without copying or selection involved.

## Graphical abstract

**Figure:**
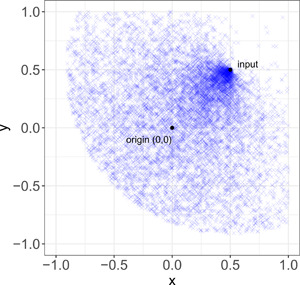

## Introduction

The richness and diversity of human cultures has been long documented by anthropologists (Benedict, 1934; Murdock, 1981; Brown, 1991; Ember et al., 1998), and biologists have observed and described behavioural traditions in several non-human species (Whiten et al., 1999; Rendell & Whitehead, 2001; Laland & Galef, 2009; Danchin et al., 2018; Aplin, 2019). In attempting to synthesise these findings and hence explain culture in a naturalistic way, a recurrent source of inspiration and insight has been the analogy with biological, Darwinian evolution (Gerard et al., 1956; Boyd & Richerson, 1985; Mesoudi, 2011; Lewens, 2015). In this perspective, culture is a population-level phenomenon consisting of items that are ‘transmitted repeatedly through social or observational learning to become a population-level characteristic’ (Whiten et al., 1999: 682) and ‘exist in identifiable form over extended periods of time’ (O'Brien et al., 2010). As such, a key question is, under what circumstances can behaviour and information that is socially transmitted exhibit some degree of population-level stability over time?

One widespread hypothesis, inspired by the Darwinian model, is that relatively stable cultural phenomena are maintained by psychological mechanisms able to copy cultural items with a sufficiently high degree of fidelity, acting in effect as a cultural inheritance system (e.g. Boyd & Richerson, 1985). This hypothesis helped to shape the study of cultural evolution (e.g. ‘Cultural learning … allows for a fidelity of transmission of behaviours and information … this fidelity serve[s] to prevent information loss … and thus … form[s] the basis for cultural evolution’; Tomasello et al., 1993: 495), and it persists to this day, including in influential overviews of the field (e.g. ‘In order for a behaviour to become traditional, it must be transmitted … without any significant loss of fidelity’; Mesoudi, 2011, p.193). Some researchers argue that the stability of cultural traditions is further supported by selective transmission biases, such as social learning strategies that cause some cultural models to be preferentially copied from over others (e.g. Laland, 2004; Kendal et al., 2018). As such, when mechanisms for high-fidelity copying are absent or play a lesser role, some other stabilising selection mechanism is present instead (see Henrich & Boyd, 2002 for a formal implementation of this approach). In sum, much existing research is built on tacit assumptions that copying and/or selection are necessary to maintain cultural stability. The overwhelming majority of formal models in cultural evolution assume that culture is realised though repeated ‘transmission’, and transmission is a process based on copying and selection.

Here we consider whether stability can emerge and persist in an evolutionary system without high-fidelity copying or selection. In other words, we examine whether the evolutionary behaviour of a system governed by copying and selection can also be obtained by other means. If it were clearly established that neither copying nor selection is necessary, that would undermine some existing foundational assumptions about the range of ways in which cultural stability can be ensured across time and space. This would in turn open the door for other frameworks for the naturalisation of culture, which might either complement or challenge the presently dominant approaches.

We focus in particular on the role of ‘convergent transformation’, in which one item causes the production of another item whose form tends to deviate from that of the original item in a non-random way (see also Claidière et al., 2018). This is a minimal and abstract notion, defined in functional terms, i.e. it specifies only a relationship between inputs and outputs. As such, convergent transformation can be realised in many different ways. We elaborate this notion conceptually – and the related notion of ‘stability’ – in the next section.

Having elaborated the key notions of stability and convergent transformation, we present three formal models, providing evidence for three main findings:

1. Stability over time at the population level can be achieved through different processes (an example of equifinality in cultural systems; Barrett, 2019). In particular, stability can emerge and be maintained by virtue of convergent transformation alone, in the absence of any form of copying or selection process.
2. As processes, high-fidelity copying and convergent transformation can be complementary in bringing about stability at the population level, but it is convergent transformation, even if weak, that drives this effect if high-fidelity copying is not also accompanied by selection.
3. While selective high-fidelity copying and convergent transformation can both produce stability at the population level (either separately or jointly), the underlying processes can be empirically distinguished through different evolutionary signatures, identified at the level of the population.

Following the models, we describe the implications of these for fundamental issues in the naturalisation of culture. In particular, we argue that while high-fidelity transmission and selection processes can serve as causes of cultural stability, they are not *necessary* causes. More broadly, we certainly do not reject the Darwinian model for culture *en bloc*, but we do argue that the possible high importance of convergent transformations, as means of maintaining cultural stability, is a disanalogy with important consequences for causal explanation.

### Cultural stability and convergent transformation

There are many characteristic examples of culturally stable phenomena. They include, for instance, children's games (some of which have remained stable for remarkably long periods of time, despite relatively fast turnover of the individuals playing them; Morin, 2015), languages (some parts of which barely change for centuries, and those parts that do change do so slowly enough for individuals of different generations to retain mutual comprehensibility), and many technologies and other artefacts (one famous example is the Acheulean hand-axe, the canonical form of which remained unchanged for hundreds of thousands of years). What these and other examples show is how traditions can remain the same over long periods of time, often in the face of ecological change. The term ‘cultural inertia’ is sometimes given to such examples, in which the items in question – not only games, parts of language or material artefacts, but also moral beliefs, categories of kinship, religious beliefs, and numerous others – exhibit a long-term, population-level stability that demands explanation (Boyd & Richerson, 1985: 56–60; Morin, 2015; Charbonneau, 2020). Inspired by examples such as these, we believe that cultural stability is best understood as a graded, population-level phenomenon, and we operationalise it as such in the models below. It is graded because traditions can be more or less stable (they can change and vary to different degrees) and can be stable over either short or long timespans, and it is a population-level phenomenon because it is best described as the product of many relatively autonomous items of different variants, with the frequency of variants changing over time (‘population thinking’: Richerson & Boyd, 2005; Claidière et al., 2018). The question is, what makes cultural stability possible in the first place? We shall suggest that convergent transformations are likely to be a crucial part of the answer.

Convergent transformation occurs whenever one item causes the production of another item whose form tends to deviate from that of the original item in non-random way. As a simple example, consider language borrowing. Words from one language are sometimes used by speakers of another, and as part of this process the words are often modified in small and unconscious ways, and not necessarily at random. Rather, many of the modifications tend in particular directions, so that the words better fit the new linguistic environment. To take just one specific example, when English words are adopted for use in Hawaiian, they are commonly transformed in ways that better fit Hawaiian phonology. English /b, f/ become Hawaiian /p/; /v/ becomes /w/; /r/ becomes /l/; /ŋ/ becomes /n/; and /t, d, θ, ð, s, z, ʃ, tʃ, dʒ, k, g/ all merge as /k/ (see Andersson et al., 2017 for many further examples). At the same time, many other sounds are not changed. The same pattern occurs with word meanings, e.g. the French ‘café’ has been adopted in many languages, but its meaning has been transformed in slightly different ways in different places, each convergent on local distinctions between different types of eateries. These examples are linguistic, but a great deal of empirical data shows clear evidence of convergent transformation in a wide range of other cultural domains (e.g. Nyhof & Barrett, 2001; Morin, 2013; Gandon et al., 2014; Miton et al., 2015; 2020; Strachan et al., 2021; see also Tennie et al., 2020 on ape culture).

Importantly, convergent transformation is an abstract notion, defined functionally. As such, it does not include specific assumptions about either (a) the psychological processes that generate cultural transmission in the first place or (b) the factors that can generate convergent transformations and influence their direction. In other words, convergent transformations can be realised in many different ways, as we elaborate below. This generality follows from how we have characterised the notion, in terms only of the relationship between input and output.

Regarding (a), convergent transformation can occur as the output of many different psychological processes. It can occur as the output of simple observation, as in the case of stimulus enhancement (in which observation of an action raises, for an observer, the salience of the object of that action), or as the output of more clearly interactive means of cultural transmission, such as active teaching, ostensive communication and others. To avoid conflating our approach with any particular means of cultural transmission, the models below use neutral language – such as ‘input’ rather than, say, ‘parental trait’ – throughout.

Regarding (b), the factors that can generate convergent transformations, and influence their direction, are many and varied. In particular, they can be both psychological and ecological (Sperber, 1996; Morin, 2015; Scott-Phillips et al., 2018). Psychological factors are cognitive competencies, preferences and dispositions, and also both currently and previously held beliefs, acquired skills, know-how, memories and other psychological phenomena held by a host (i.e. a biological individual) that affect whether and how a cultural item is processed by that host. Ecological factors are, in contrast, those factors in the shared local environment that are relevant to cultural dynamics. They include the biological and physical environment external to the organism (food and materials) and also behaviours and artefacts, including public representations such as speech, writings and ritual performances, through which people interact with one another. The empirical impact of both types of factors on processes in cultural evolution has now been experimentally documented many times (for psychological factors see e.g. Kalish et al., 2007; Miton et al., 2015; Scott-Phillips, 2017; for ecological factors see e.g. Schillinger et al., 2014; Miton et al., 2020).

As such, convergent transformation is a general notion that is not equivalent to any of the notions currently common in the cultural evolution literature. It is different from transmission biases, because transmission biases act as a selection process (some content is more likely to be transmitted than others), while convergent transformation is a source of variation (some content is more likely to be altered in some ways rather than others). Convergent transformation is also different from random copying error, or cultural ‘mutation’, because with convergent transformations some ‘mutant’ forms are more probable than others, and not necessarily in the direction of greater adaptiveness. In this sense, convergent transformation is somewhat analogous to mutation biases in biological evolution (see Yampolsky & Stoltzfus, 2001; Stoltzfus, 2006; Stoltzfus & Yampolsky, 2009). Finally, convergent transformation is different from guided variation, with which it has been sometimes compared (Richerson & Boyd, 2005; Acerbi & Mesoudi, 2015). The main difference is that guided variation is used to describe psychological processes of individual learning leading (‘guiding’) to fitness enhancement (Richerson & Boyd, 2005), whereas convergent transformation is a stochastic process that can arise in many ways, and as such need not entail hill-climbing, and need not depend on intelligence, insight or innovation. (Note that some accounts of guided variation downplay its directional aspect, making them, in this respect, more similar to convergent transformation, e.g. Mesoudi, 2011; Acerbi & Mesoudi, 2015.)

The notion of convergent transformation can thus contribute to the development of models of cultural evolution that, while retaining their basic evolutionary nature, are more general than models based on a stricter adherence to the Darwinian approach (Claidière et al., 2014a, b). Specifically, we present a series of stochastic simulations that compare and contrast the effects of convergent transformations with the effects of other hypothesised causes of cultural stability, in particular those inspired by the comparison with biological evolution such as faithful transmission (copying), random error and transmission biases. In this way our models complement some other, ongoing research agendas, namely those that examine how convergent transformation and selection can interact with one another (Claidière & Sperber, 2007; Claidière, 2009; Claidière et al., 2018), and how convergent transformation influences the subsequent co-evolution of culture and cognition (Kirby et al., 2007; Griffiths et al., 2008; Thompson et al. 2016).

More generally, formal models serve a range of scientific, epistemic and philosophical purposes, from abstract ‘proof-of-concept’ models that specify and test the internal logic of verbal argument, to models that simulate specific empirical processes, sometimes generating quantitative predictions as a result (Frigg & Hartmann, 2020). Our Models 1 and 2 aim at the first of these goals (see Servedio et al., 2014 on the importance and utility of proof-of-concept modelling). That is, they test the internal logic of previous verbal and philosophical arguments about the cognitive foundations of culture and cultural transmission (see also Boyd & Richerson, 1987; Lewens, 2015). In Model 3, we extend our approach to generate a rubric for identifying cases of cultural transmission where convergent transformation is likely to have played a significant role, based on its signature in empirical records.

## Methods

We first describe the general methods that apply to all our simulations. Code for all models is available in an Open Science Framework repository at https://osf.io/yncws/

We consider populations of items in a variation space (inspired by Sperber, 1996: chapter 5) with each axis representing a continuous arbitrary feature of a cultural item. This could be, for instance, the size and width of an arrow-head (as in e.g. O'Brien et al., 2016); different features of rhymical structure in music (as in e.g. Miton et al., 2020; Strachan et al., 2021) or the form and meaning of a word (as in many language evolution experiments, see Tamariz, 2017 for a review). We consider a population of *N* items. (In Models 1 and 2, *N* = 100; in Model 3, *N* = 10, 100, 1000.) At the beginning of each simulation, items are randomly placed in a continuous bidimensional (square) space with coordinates in the range [−1, 1]. At each timestep, the original population of items is replaced by a population of new items of equal size *N*. The location of each item at time *t* + 1 is determined by applying a stochastic transformation function that takes the location of an item at time *t* as input, and we study the evolution of the location of these items over time.

The process proceeds through three ordered stages.

**Figure 1.**
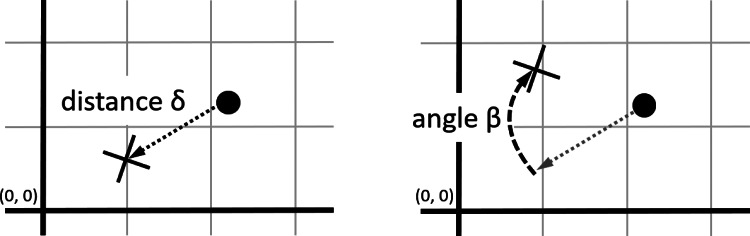
Transformation function. The input is depicted with a filled circle and the output with a cross. The transformation function determines a distance and an angle (see main text). The distance, *δ*, is measured absolutely (left panel), whereas the angle, *β*, is measured relative to a straight line between input and origin (right panel).

**Figure 2.**
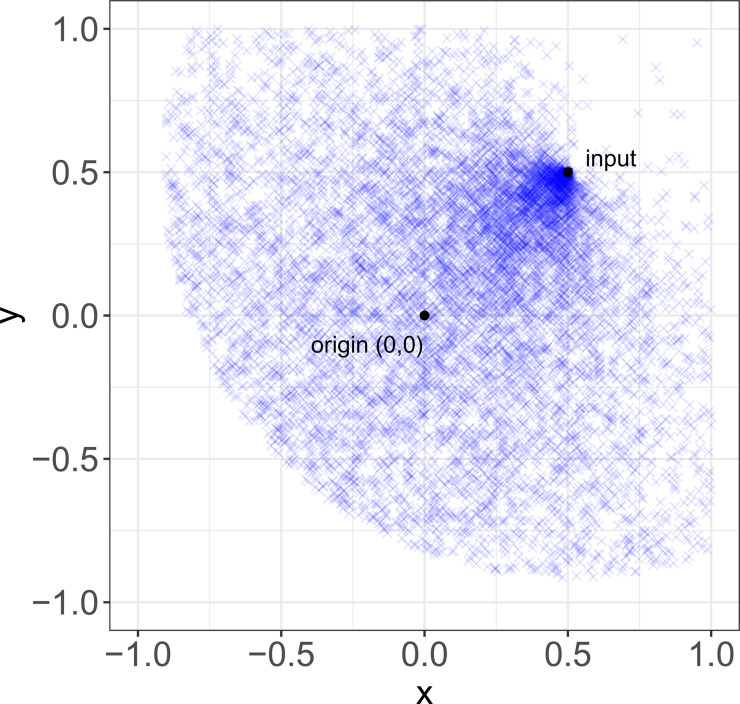
Convergent transformation. Representative distribution of items (*N* = 10,000) after convergent transformation, given an input at location (0.5, 0.5).

1. *Sample population*. For each item at *t* + 1, the population at *t* is sampled in one of two ways, either random or biased:
  - *Random sampling.* One item from the population at time *t* is sampled at random and used as input for the transformation function.
  - *Biased sampling.* Two items from the population at time *t* are sampled at random, and whichever is closest to the origin (0, 0) (the centre of the space) is used as input for the transformation function. This effectively represents a selection process in which variants closer to the origin are fittest. The overall space can be understood, in this case, as a continuous, smooth fitness landscape with a single peak at the origin. (An alternative approach, more similar to classical model in population genetics, could be to sample *N* items from the previous generation by drawing each item with a probability proportional to their distance from the origin.)
2. *Apply transformation function*. New items undergo one of two transformation functions, either random or convergent. For each new item, both its distance from, and angle relative to, its input is determined by probability distributions, as follows (see also Figure 1):
  - *Random transformation*. The location of the item at *t* + 1 is equal to the location of its input modified by a distance *δ*_r_ and an angle *β*_r_:
    - *δ*_r_ is drawn from a lognormal distribution with *σ* = 1 and *μ* = 0, rescaled to a range [0, *k*], where *k* is a parameter of the simulation; *k* can be thought of, intuitively, as the maximum possible ‘copying error’ (but this gloss should not be interpreted as entailing any assumption that cultural transmission entails cognitive processes of ‘copying’; see above).
    - *β*_r_ is drawn from a uniform distribution in the range [−*π*, *π*] radians with 0 oriented towards the origin. Because this distribution is uniform, the angle between an item at time *t* + 1 and its input at time *t* is random.
  - *Convergent transformation*. The location of the item at time *t* + 1 is equal to the location of its input at time *t* modified by a distance *δ*_c_ and an angle *β*_c_, as described below:
    - *δ*_c_ is drawn from a uniform distribution in the range [0, 2*d*], with *d* being the distance of the input to the origin. This means that the distance between an item and its input is a function of the distance between the input and the origin. The closer an input is to the origin, the smaller the distance between it and the item at *t* + 1 will be.
    - *β*_c_ is drawn from a normal distribution in the range [−*π*, *π*] radians with *σ* = 1 and *μ* = 0, and with 0 oriented towards the origin. Because this distribution is normal, the direction between an item at time *t* + 1 and its input at time *t* is not random. Instead, items are most likely to be located closer to the origin rather than away from it.
  Where the final location of an item results out of the boundaries of the variation space, the transformation function is repeated until the item falls within it. Different ways to handle these occurrences do not change our results.
  These various functions reflect empirical aspects of the different processes by which stability might be achieved. For random transformations, *δ*_r_ is defined as a lognormal distribution to reflect the idea that, while most copying errors are small, exact replication is a marginal case, and *β*_r_ is defined as a uniform distribution to reflect the idea that ‘copying errors’ are undirected. These two ideas are both common in the cultural evolution literature. For convergent transformation, *δ*_c_ is defined in terms of *d* to reflect the idea that similarity between items and their inputs is not a fixed quantity, as it usually is with copying errors, but traits tend to transform more or less over time in virtue of their properties (Sperber, 2000; Mesoudi & Whiten, 2004; Scott-Phillips, 2017), in our case represented by their location in the variation space. To help understanding the effect of convergent transformation, Figure 2 shows a representative distribution of items after transformation, given an input at location (0.5, 0.5).
3. *Iterate and measure.* Items of time step *t* are removed, and the items from time step *t* + 1 serve as the inputs for the next time step. Once the location of each item at *t* + 1 is set, we measure two aspects of the evolution of the system: stability and similarity.
  - *Stability*. We take two types of measures relevant to stability: (a) change in geometric centre of the population i.e. the mean trait value; and (b) spread of the population. Mean trait value is the most common measure in evolutionary biology (Hartl & Clark, 1997). Correspondingly, it is often the only measure of stability used in studies of cultural evolution. However, (a) and (b) are both important as they can vary independently, and both are relevant. For example, in cases of disruptive selection, or stabilising selection, the geometric centre could remain the same while the spread would change. For instance, polarisation in political views may not be captured by looking only at the mean expected value: to capture such changes properly, spread must also be measured. As such, ignoring either measure could lead to missing some important forms of evolutionary change. Stability is best understood in light of both measures.
    - We measure the change in geometric centre over time by calculating the centroid of the population and then calculating the Euclidean distance between the centroids at two different intervals. These intervals are either 1 time step apart or 100 time steps apart.
    - The spread of the population is a measure of the clustering of the items at a given time step. This is defined as the average distance of all items from the centroid of the population (the second moment of our distributions).
  - *Similarity.* The degree of similarity between an item and the input it was produced from is measured as the Euclidean distance between them. Similarity at the level of the population is then the mean of these distances for all items in the population. This measure is used in Model 3 only, where we investigate whether different possible causes of stability have different evolutionary signatures at the level of the population

## Results

### Stability without copying or selection (Model 1)

We first investigate the different conditions under which cultural stability obtains, or not. We compare five conditions (*N* = 100 in all cases), as below. In this way we are able to directly compare the effects of different sampling and transformation functions on the behaviour and, in particular, the stabilisation of the evolving population.

- *Baseline*. The location of all items at each time step is determined wholly at random, i.e. there is no sampling or transformation, and hence no relationship between the population at time *t* and time *t* − 1.
- *Replication*. Random sampling with no transformation.
- *Unbiased*. Random sampling with random transformation, under three distinct values of *k* (*k* = 0.01; 0.1; 0.5).
- *Biased sampling*. Biased sampling with random transformation, under three distinct values of *k* (*k* = 0.01; 0.1; 0.5).
- *Convergent transformation*. Random sampling with convergent transformation.

**Figure 3.**
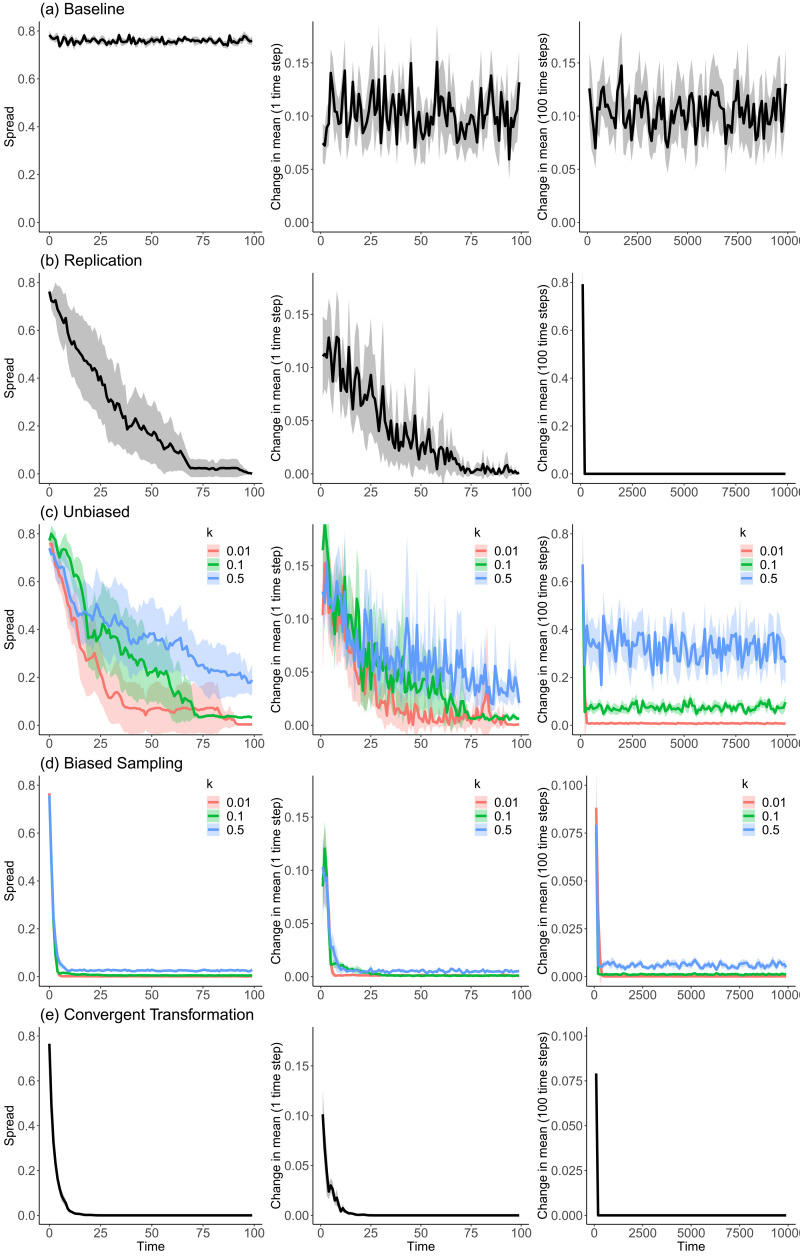
Output of Model 1. Each row represents a different set of 10 simulations, and each column measures a different aspect of cultural stability. Simulations from top to bottom: (a) baseline; (b) replication; (c) unbiased; (d) biased sampling; (e) convergent transformation. Measures of stability, from left to right: (left) spread; (centre) change in geometric centre across 1 time step (simulation ran for 100 time steps); (right) change in geometric centre across 100 time steps (simulation ran for 10,000 time steps). All results are averaged over 10 runs of simulations. The shaded area shows standard deviations. In all conditions *N* = 100.

Figure 3 summarises the behaviour of the model under the different conditions described above. All of the results presented are an average of 10 runs of simulations. Videos of representative runs of simulations (b)–(e) are provided in the Supplementary Material (videos SM1–SM4).

The results highlight two particularly relevant aspects of cultural stability. First, a population under convergent transformation alone (Figure 3, row e) achieves stability. Both spread and change in geometric centre decrease asymptotically towards 0, and remain there. These results are robust to changes regarding the multiplier of *d* used to calculate the range of the uniform distribution from which *δ*_c_ is drawn, and the variance of the normal distribution from which *β*_c_ is derived (see Supplementary Information). The result shows that neither high-fidelity transmission nor selection are necessary for stability: convergent transformation can be sufficient on its own. While cultural evolutionists would agree that stability can be brought about by other forces, in practice many models assume that high-fidelity copying and selection are the necessary engines behind it. This result thus undermines assumptions that have been and continue to be influential in the cultural evolution literature, as we detailed in the Introduction. Arguments to this effect have been made verbally, but formal demonstration makes the rationale explicit and facilitates direct comparisons (such as those we present in Models 2 and 3).

Second, a population under random transformation achieves stability only when coupled with a biased sampling process. In ‘Unbiased’, in which sampling is random, there is short- but not longer-term stability, even when *k* is extremely low, such as in *k* = 0.01 (results not shown). Indeed, the geometric centre drifts through time: although there is very little change in geometric centre between two close time steps (1 or 100), over the long term small changes in mean trait value add up, producing drift as an outcome. In other words, high-fidelity transmission produces long-term stability only when coupled with selection. This is, we note, similar to a standard result in population genetics, and employed in molecular evolution, where the observations of abnormal evolutionary stabilities in molecular sequences are used as evidence of the effects of selection (see Millstein, 2002 for discussion).

### Mixing random and convergent transformation (model 2)

In Model 1, convergent transformation was sufficient to produce stability on its own. In reality, we might expect high-fidelity copying and convergent transformation to act on different items to various degrees. Indeed, the relative importance of high-fidelity copying and convergent transformation is debated (e.g. Claidière & Sperber, 2007; Acerbi & Mesoudi, 2015). To clarify these issues, we develop a model mixing both types.

Specifically, we constructed a model with random sampling and a function that determines which type of transformation – random or convergent – will occur. The probability that the transformation will be convergent is equal to 1 − *d^α^*, where *d* is the Euclidean distance between the input and the origin (scaled between 0 and 1) and *α* is a parameter of the model ranging between 0 and ∞. Otherwise, the transformation is random. Thus, the closer an input is to the origin, the more likely it is that transformation is convergent. A high *α* increases the overall probability that an item is transformed directionally instead of randomly. As *α* decreases, so does the overall probability that an item is transformed in a convergent rather than random way. *α* = 0 reduces to condition (c) in Model 1 (‘Unbiased’), and *α* =∞ reduces to condition (e) (‘Convergent transformation’). Note that other functions relating *α* and *d* to the probability that a transformation will be convergent could be employed to model specific empirical phenomena.

**Figure 4.**
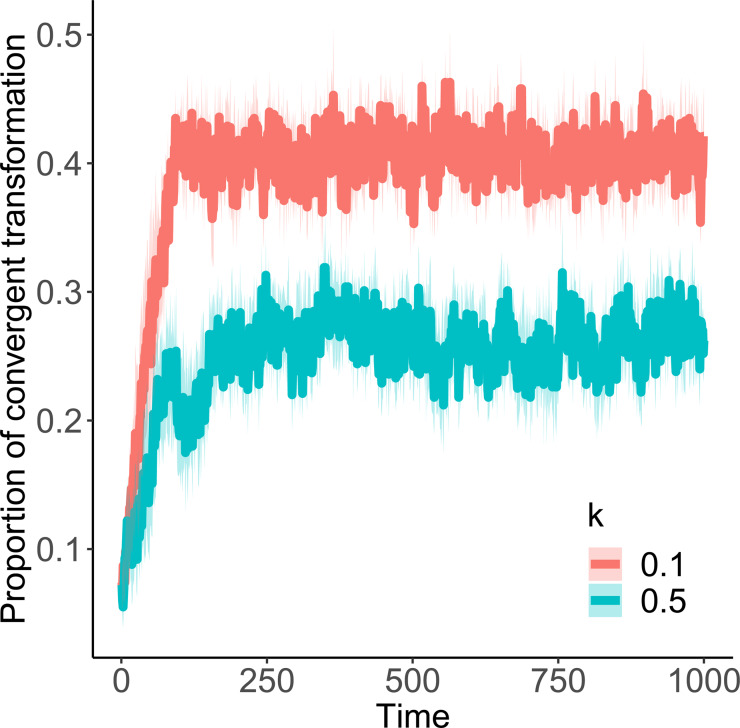
Proportion of items that, at each time step, are subject to convergent transformation in Model 2, with *α* = 0.1 and different copying fidelity (*k* = 0.1; 0.5). Results are averaged over 10 runs of simulations, all with *N* = 100. The shaded area shows standard deviations.

The results show that, in situations where there is both convergent transformation and high-fidelity copying (intended here as a property of the transformation mechanism itself, or ‘propensity fidelity’ in Charbonneau, 2020), these two factors end up reinforcing one another to secure stability at the location where convergent transformation alone (and not copying alone) would have stabilised the population. This occurs even with high values of *k* (e.g. *k* = 0.5), where unbiased copying alone would not produce a stable population (see Figure 3c). Interestingly, the proportion of items that undergo convergent transformation is *higher* when *k* is lower than when it is higher. That is, convergent transformation, keeping *α* constant, is *more* common when copying ‘errors’ are smaller (Figure 4). This is because once an input is brought within the vicinity of the origin, future items are more likely to remain within that vicinity, and thus subject to convergent transformation, when *k* is low than when it is high. In short, although convergent transformation and high-fidelity copying work together to secure stability, it is convergent transformation that drives the effect.

The findings of Models 1 and 2 open up questions of high relevance to the empirical study of culture. Since stability can be achieved in more than one way – by high-fidelity copying and selection, or by convergent transformations – how might we differentiate between these different possible causes? Is there a way to identify, in empirical records, whether one or the other of the different processes we have modelled is in fact at play in a given case? Model 3 investigates these questions.

### Evolutionary signatures of different causes of stability (Model 3)

Here we investigate whether different possible causes of stability have different evolutionary signatures at the level of the population. To do this, we study how a population with a limited spread and far from the centre of the variation space (i.e. far from the equilibrium point) evolves over time. To cluster the population, we started the simulation by first locating the items in one of the four corners (chosen at random, with a 0.8 distance from the origin for both *x* and *y* coordinates, and randomly distributing the items within 0.05 distance from that point). We ran conditions ‘Biased sampling’ (with *k* = 0.1; 0.5) and ‘Convergent transformation’ with this new starting setup. To examine how populations evolve towards the origin, we track similarity between items and their inputs. As in previous models, *N* = 100. We also investigate the impact of population size, and we repeat the above process for three different population sizes (*N* = 10; 100; 1000). We measure how many time steps it takes for the populations to reach a stable state, which we operationalise as a change in geometric centre at each time step less than or equal to 0.01.

#### Fidelity

**Figure 5.**
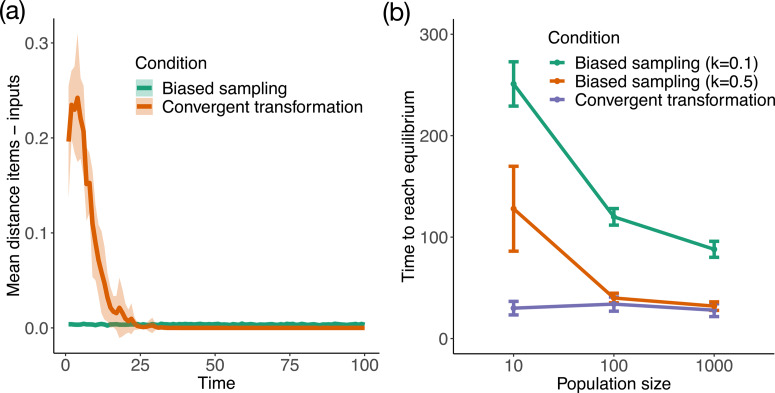
Population-level similarity (a) and effects of population size (b) in Model 3. (a) Similarity between items and their inputs. ‘Biased sampling’ with *k* = 0.1 and ‘Convergent transformation’ as in Study 1. Results are averaged on 10 runs of the model. The shaded area shows standard deviations. In both conditions *N* = 100. (b) Time to reach equilibrium for different population sizes. Measured for ‘Biased sampling’ at two different levels of k (*k* = 0.1; 0.5) and for ‘Convergent transformation’. Results are averaged on 10 runs of the model. Bars show standard deviations.

In ‘Biased sampling’ the mean distance between items and inputs (or ‘episodic fidelity’ in Charbonneau, 2020) is relatively low (depending on *k*) and remains so throughout the simulation (see Figure 5a). This is due to constant rate of random transformations, which derive from the assumption that transmission processes possess a specific degree of fidelity. This is how it is characterised in the theoretical literature, and implemented in other models (see Charbonneau, 2020 for critical discussion). In contrast, in ‘Convergent transformation’ we observe at first a high distance between items and their inputs (i.e. a low degree of similarity) and then a rapid decrease of distance (see Figure 5a). This is because the expected degree of similarity is not fixed, but instead depends on the specific location of the input. This represents the idea that that the degree of fidelity by which a cultural trait is transmitted is not an intrinsic property of some underlying transmission process, but it can depend on the specific content under transmission (Acerbi & Mesoudi, 2015; Charbonneau, 2020; Charbonneau & Bourrat, 2021). In particular, the further from the origin an item's input is, the less similar we can expect the item to be from the input. Qualitatively, in the ‘Biased sampling’ case the population moves together, in small steps, until it reaches the origin, producing a gradual evolution akin to a hill-climbing behaviour. In contrast, in the case of ‘Convergent transformation’ the population rapidly converges on the origin, in what can be described as a ‘jumping’ behaviour, with little effect of cultural inertia. See videos SM5 and SM6. Notice that, while smaller values of *d* could make the ‘Convergent transformation’ case appear qualitatively similar to a hill-climbing behaviour, large values of *k* would *not* produce a ‘jumping’ behaviour for ‘Biased sampling’, since the population will not be subsequently stabilised in the origin.

#### Population size

By varying population size, we observe that the two different conditions show very different sensitivities to population size (Figure 5b). There is no evidence of sensitivity to population size in ‘Convergent transformation’, but there is in ‘Biased sampling’, with larger populations taking less time to reach the same degree of stability as smaller ones. This dependency on population size occurs because biased sampling is, fundamentally, a sorting process dependent on sample size: it is more likely to sample one item closer to the origin in a large population than in a small population.

In sum, we identify two evolutionary signatures that can distinguish between different causes of cultural stability. First, there are qualitative and quantitative differences in the behaviour of the population as it converges on a stable form. Specifically, the similarity between items and respective inputs is constant with biased sampling, but variable with convergent transformation. Second, there are differences in sensitivity to population size. Specifically, there is sensitivity to population size only in the case of biased sampling, but not in the case of convergent transformation.

## Discussion

In the Introduction we said that one important motive for examining the population-level effects of convergent transformation was to address the question of whether the evolutionary behaviour of a system governed by copying and selection can be obtained by other means, opening the door for alternative, yet potentially complementary frameworks. Many distinct research traditions, across evolution, psychology and anthropology, have either argued or assumed that cultural stability, whether over shorter or longer timespans, requires psychological mechanisms (e.g. imitation) capable of copying cultural items with some high degree of fidelity (e.g. Mesoudi, 2011; Legare & Nielsen, 2015; Henrich, 2015). Are they correct to do so?

We have shown that the observation of cultural stability does not necessarily imply the existence of copying mechanisms. In particular, Model 1 shows that stability can emerge in an evolutionary system by virtue of convergent transformation alone, in the absence of any form of copying or selection process. This effect is robust to a sensible range of parameter values and is not idiosyncratic. It follows that the emergence and long-term persistence of cultural traditions does not necessarily require any processes of transmission whose proper (evolved) function is high-fidelity transmission of cultural information. High-fidelity copying is therefore but one of several factors that can ensure intergenerational stability in an evolutionary system (see also e.g. Griffiths et al., 2008; Acerbi et al., 2012; Dean et al., 2014). Cultural traditions can also emerge and remain stable as a consequence of any social process – of which there are many – that produces convergent transformation.

Notice that the way we implemented convergent transformation in our model cannot be equated with a form of (even imprecise) copying. While it is true that the items taken as inputs are in a causal relationship with the items produced as outputs, this is a necessary condition for any population to evolve from a time *t* to a time *t +* 1 (contrast with Model 1 condition (a) where there is no such causal relation). However, convergent transformation, in our model, differs from a process of copying, as it is usually modelled in cultural evolution, in at least three respects. First, it is context sensitive, i.e. the amplitude and directionality of expected change depends on the value (position) of the inputs, while copying is context insensitive: copying occurs in the same way independently of what the input is. Second, the modifications are not random, i.e. the final position of the items tends to diverge from the position of the inputs in a directed manner, while copying errors are random. Third, for copying, transformations (copying errors) tend to be modelled as small, in a mutation-like manner. If a copying process is to be successful, the copy will show little change from the input it was created from. In contrast, in convergent transformation, modifications can be large (jump-like), depending on the position of the input (again, because of content sensitivity).

These points have previously been argued for mostly in a verbal way (e.g. Sperber, 2000; Claidière & Sperber, 2010; Morin, 2015; Charbonneau, 2020; Love & Wimsatt, 2019; for other formal treatments see Claidière & Sperber, 2007; Claidière, 2009; Claidière et al., 2018; Morgan & Thompson, 2020). Here, following a conceptual elaboration of convergent transformation, we have subjected these previous arguments to formal probing in highly general ways, made them comparable with concurrent models and found the arguments robust. We are thus presenting a precise challenge to many existing assumptions about the extent of the analogy between biological and cultural evolution (see also e.g. Dennett, 2006; Claidière & André, 2012; Lewens, 2015; Driscoll, 2017; Nettle, 2020). Some loosening of the Darwinian analogy, greater than most existing frameworks presuppose, is justified.

At the same time, models based on convergent transformation still maintain the desirable features of the models based on the standard analogy. In particular, they are populational, and can still realise a form of mindless variation-introduction, in which properties of the system that are observable at the population level need not be instantiated at the individual level (Richerson & Boyd, 2005; Claidière et al., 2014a, b). That is, even without a strict Darwinian framework, cultural evolution can still produce a ‘collective brain’ that outsmarts single individual brains (Muthukrishna & Henrich, 2016; Dennett, 2017). In addition, convergent transformation allows in principle for a very large suite of factors that can influence the population-level outcomes. As we discussed in the Introduction, our models do not implement specific psychological processes: while universal cognitive tendencies, often investigated in evolutionary psychology, can and do exemplify possible factors that guide convergent transformation, many other factors, including ecological ones, can also do so (see e.g. Miton et al., 2020 for experimental demonstration).

A further important issue is the combined effect of high-fidelity copying and convergent transformation when they act together in an evolutionary system (Model 2). Previous models have shown that, when copying is biased, and the biases act in the same direction of convergent transformation, the effects will reinforce each other (Henrich & Boyd, 2002; Claidière et al., 2018). When instead the effects of copying biases and convergent transformation are in opposition, the end state depends on the relative force of the two (Claidière & Sperber, 2007; Claidière et al., 2018). Given that, in our model, biased sampling and convergent transformation acted in the same direction, we analysed, with Model 2, the case of high-fidelity copying with *unbiased* sampling and convergent transformation. Intuitively, it might be expected that faithful unbiased copying will ‘lock’ items in a different point of the variation space with respect to where they would end if convergent transformation operated alone (i.e. the attractor point), and that this effect would be stronger the more precise the copying. However, the results show that, the more faithful the copying is, the *stronger* the effect of convergent transformation. Put simply, unbiased copying reinforces the only directional mechanism present, namely convergent transformation, making items close to the origin more stable than what they would be with less faithful copying. This suggests that convergent transformation, even when of low magnitude, can counteract – or might potentially even dominate – the effects of other factors with shifting directionality (such as, for instance, model-based social learning strategies; Kendal et al., 2018).

Our results also identify two evolutionary signatures of different possible sources of stability in an evolutionary system, given that populations start far from the equilibrium point and need to explore the space to reach it (Model 3). The first signature concerns different levels of similarity while a population is undergoing change. Specific and clear examples can be widely seen in the experimental literature on language evolution, which consistently shows the pattern observed for ‘convergent transformation’ in our Model 3. Levels of intergenerational similarity are at first low, when the languages are unstructured and relatively inefficient, and later high, once the languages have evolved structure and greater levels of communicative efficiency. Similar points apply to several other experimental datasets too, across a range of different cultural domains (Mesoudi et al., 2006; Lewandowsky et al., 2009; Miton et al., 2015; Ravignani et al., 2016; Claidière et al., 2018). Our second evolutionary signature is differential sensitivity to population size. Many recent studies investigate the relationship between population size and the cumulative complexity of cultural items, in particular technology (Henrich, 2004; Powell et al., 2009; Querbes et al., 2014). The hypothesis here is that larger populations increase rates of technological progress, because larger population ensure lower risks that cultural traits become rare and are lost. Our study adds to this literature an important additional finding about the relative rates of convergence upon new cultural items. More generally, where the stabilisation of a cultural item in a population is influenced by the size of that population, this may be interpreted as (partial) evidence that biased selection and copying plays a role, and conversely where there is no such relationship, convergent transformation is likely to be more important (Acerbi et al., 2017). A similar pattern has been recently highlighted in a comparable model (Mesoudi, 2021), suggesting that the analysis of the relationship between the effect of demographic features on cultural dynamics and the respective role of selection and convergent transformation represents a fruitful avenue for future empirical studies.

More broadly, our simulations also contribute to the formal modelling of cultural attraction. At root, Cultural Attraction Theory argues that convergent transformations are a common and ordinary feature of human interaction, and hence that cultural fidelity and cultural stability are best seen as emergent properties at the population level, with many possible local causes: ‘Cultural causality is promiscuous’ (Sperber & Claidière, 2006: 22; see also e.g. Sperber, 1985, 1996; Claidière & Sperber, 2007; Claidière et al., 2014a, b; Morin, 2015; Heintz, 2017; on cultural fidelity see Charbonneau, 2020). Our models make these claims more precise, and hence advance recent debate about whether Cultural Attraction Theory might provide a productive framework for studying culture from a naturalistic perspective (e.g. Acerbi & Mesoudi, 2015; Sterelny, 2017; Buskell, 2017; Scott-Phillips et al., 2018).

One question is why, if convergent transformations are so ubiquitous, there is still cultural change and variation. Indeed, our models show how convergent transformations can quickly cause populations to converge and then be stable in perpetuity, but if that is correct, why do cultural phenomena still change over time, even if just gradually so? In answering this question, two features of convergent transformation are especially important. First, convergent transformations are stochastic (and cultural attraction is hence probabilistic and not deterministic; see Claidière & Sperber, 2007). Second, in any given case the factors relevant to transformations can be many, and the mix can vary from case to case. Our models implement just one factor in all cases, causing the items in the population to converge to one particular point in the space. However, in most real-world cases the factors that contribute to convergent transformation are multiple and they can themselves vary in time. Together with a degree of stochasticity, this diversity maintains variation in the population, potentially at high levels. In consequence there will be, in any moderately complex system based on convergent transformation, a high sensitivity to initial conditions, and persistent change alongside the stability that we have focused on here.

More broadly, we note that by virtue of their generality (see in particular the section ‘Convergent transformation’), our models can be extended in many ways, to study cultural dynamics of many different types. Here we highlight three possibilities. First, in our models, the convergent transformation function is oriented towards one single point in the space (the origin), but the model can be easily adjusted to include multiple points of convergence. Similarly, the functions for convergent transformation and biased sampling are presently both oriented towards the same point in the space, but this can be easily altered by re-defining one or the other to be oriented to some other point. Finally, at present, the function that determines the distance covered by convergent transformation is defined in such a way that the closer an item is to the origin, the shorter the distance is, and vice versa. This could be modified in various ways, tailoring the model to specific issues.

## Supplementary material

For supplementary material accompanying this paper visit https://doi.org/10.1017/ehs.2021.47. click here to view supplementary material

## Research transparency and reproducibility

Code for all models is available in an Open Science Framework repository at: https://osf.io/yncws/
