# Supplementary material for: Culture without copying or selection
Source: Evol Hum Sci. 2021 Nov 4;3:e50. doi: 10.1017/ehs.2021.47 (PMC10427323; doi:10.1017/ehs.2021.47)
Supplement: Supplementary file 1 [file S2513843X21000475sup001.zip › ESM_text.docx]

**Supplementary Information for:**

**Culture without copying or selection**

Alberto Acerbi, Mathieu Charbonneau, Helena Miton, Thom Scott-Phillips

To test the robustness of convergent transformation results – Model 1 (e) –, we systematically varied the multiplier of *d* used to calculate the range of the uniform distribution from which δ*_c_* is drawn, and the variance (σ) of the normal distribution from which β*_c_* is derived. Both parameters are varied from 0.2 to 4, which steps of 0.2. As in the simulations described in the main manuscript, we used two measures of stability: the spread and the change in the geometric centre of the population over time (calculated at intervals of 1 and 100 time steps).

For spread and change in geometric centre with 1 time step interval, we calculated the outputs averaging the last 100 time steps of simulations running for 1,000 time steps. For change in geometric centre with 100 time step intervals, we run simulations for 10,000 time steps, and averaged the measured in the last 1,000 time steps.

The results are summarised in Figures 1SM, 2SM, and 3SM below and they highlight a similar pattern. Increasing the multiplier of *d* implies that items can land farther from their inputs. Increasing the variance of the normal distribution from which β*_c_* is derived implies flattening the distribution, thus widening the angle where items are likely to fall with respect to the origin. Both modifications result, as expected, in the transformations being *less* convergent. For example, if the multiplier of *d* is 4, it is likely that items will be farther from the origin than their inputs, resulting mostly in *non*-convergent transformations. For a sensible space of parameters where transformations are effectively convergent (excluding the top-right corners of Figures 1SM, 2SM, and 3SM) our main results regarding stability prove robust.

**Figure 1 SM.** Robustness check for convergent transformation - spread. Each point represents the average spread in the last 100 time steps of simulations running for 1,000 time steps. The red square represents the parameters used for the main simulations. In all conditions *N* = 100.

**Figure 2 SM.** Robustness check for convergent transformation – change in centre, 1 time step. Each point represents the average change in the population centre calculated over the last 100 time steps of simulations running for 1,000 time steps. The red square represents the parameters used for the main simulations. In all conditions *N* = 100.

**Figure 3 SM.** Robustness check for convergent transformation – change in centre, 100 time steps. Each point represents the average change in the population centre calculated over the last 1,000 time steps of simulations running for 10,000 time steps. The red square represents the parameters used for the main simulations. In all conditions *N* = 100.
